# The Taxonomic Significance of ducts in the corolla lobes of *Vernonia* (Vernonieae: Asteraceae)

**DOI:** 10.3897/phytokeys.58.7009

**Published:** 2016-01-12

**Authors:** Harold Robinson, Stanley Yankowski

**Affiliations:** 1 Department of Botany, MRC 166, NMNH, P.O. Box 37012, Smithsonian Institution, Washington, DC. 20013-7012, USA

**Keywords:** Ducts, corolla lobes, Vernonia, Vernonanthura, Trepadonia

## Abstract

The multiple longitudinal ducts in the corolla lobes found in the closely related genera *Vernonia*, *Vernonanthura* and *Trepadonia* are microscopically studied and illustrated. The lack of such ducts in the two South American species that have until now been retained in *Vernonia* indicates that they should probably be excluded from the genus.

## Introduction

The generic concept of the genus *Vernonia* was simple and uninsightful for over 150 years from the time of it’s inception in Schreber (Gen. 2: 541. 1791, nom. cons.), with the eastern North American type *Serratula noveboracensis* L., type cons. *Vernonia* was the core genus of the tribe Vernonieae, having the combination of capillary pappus and non-liguliform florets that were common to all core genera of the Asteraceae during that time. Some segregates were named, such as *Baccharoides* Moench. and *Gymnanthemum* Cass., in Africa, *Eremosis* Gleason in Mesoamerica and *Critoniopsis* Sch.Bip. in South America, but these were not generally recognized at the generic level.

The concept of *Vernonia* began to change in the last 30 years as is partially summarized in the treatments by Robinson for the Paleotropical (1999a) and the American members (1999b) of the tribe. The altered concept reduced the genus *Vernonia* to a restricted group of species mostly directly related to the type species *Vernonia noveboracensis* (L.) Michx., a group with eleven species in eastern North America, ca. 5 species in the highlands of central Mexico, and one eastern North American species reaching the Bahamas. Until now, two species in South America have been retained in *Vernonia*. The genus consisted of species having a cymiform inforescence with seriate “scorpioid” cymose branches deflected at the nodes, glanduliferous anthers, a somewhat rhizomiform rootstock and echinate weakly sublophate pollen. Closest relatives were considered to be members of the genus *Vernonanthura* H.Rob. with ca. 68 species in tropical America, and *Trepadonia* H.Rob. with two species on the eastern side of the Andes in Peru and western Brazil. *Vernonanthura* differed most notably by the more pyramidal inflorescence with cymiform branches and an often tuberous rootstock, and *Trepadonia* was scandent with widely spreading lateral branches in the inflorescence. The comparative close relationship between *Vernonia* and *Vernonanthura* was reflected fully in the DNA sequence studies of Keeley et al. (2007).

**Figure 1.**
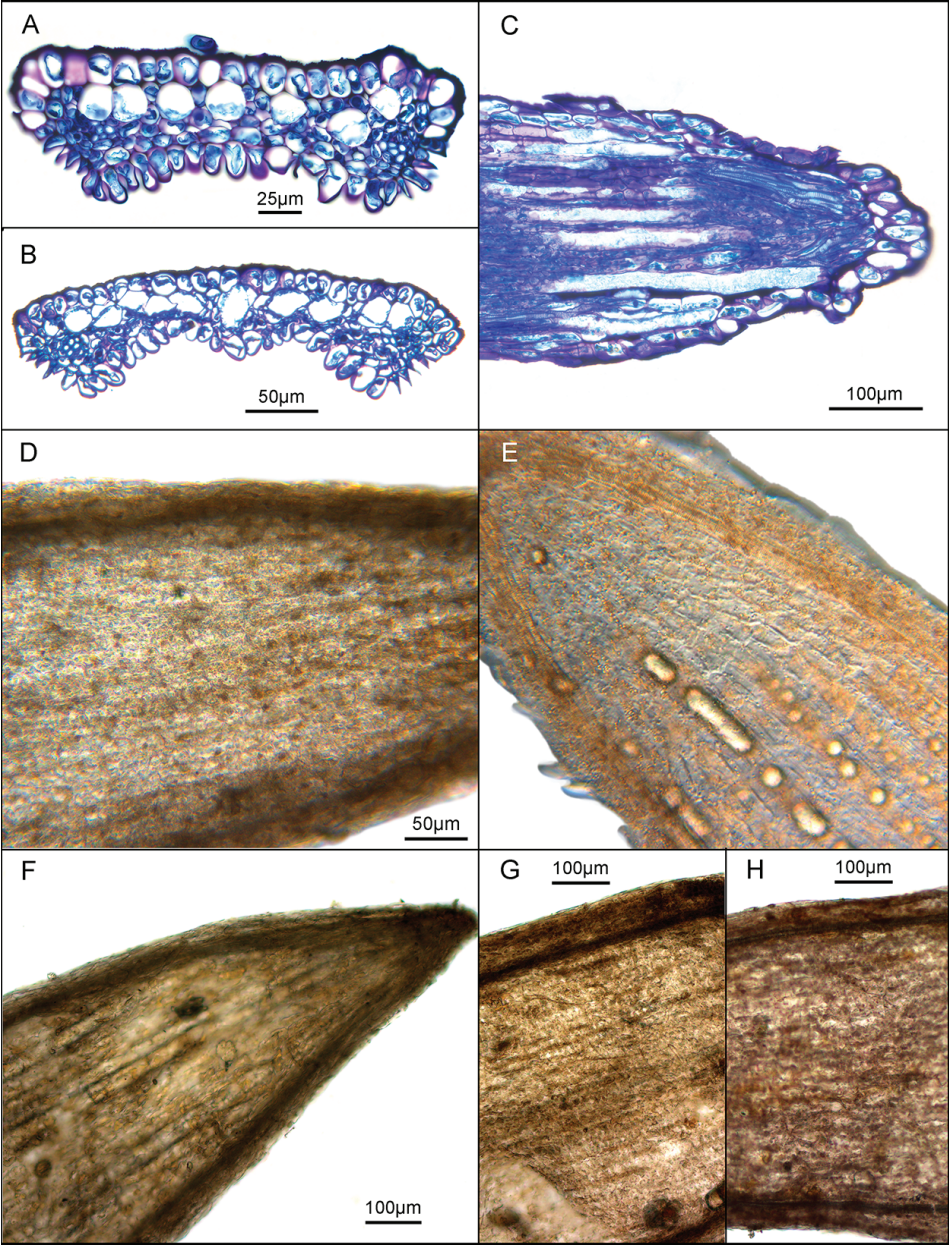
**A–D**: **A**, **B**, *Vernonia noveboracensis* cross-sections of corolla lobes, outer surface upward **C** longitudinal section of corolla lobe showing multiple ducts **D** corolla lobe from specimen in preservative showing only poorly defined partitions between ducts **E** *Vernonia angustifolia*, corolla lobe from freshly collected specimen showing fresh resin in ducts **F** *Vernonanthura brasiliana*, ﻿corolla lobe from dried herbarium specimen showing solidified contents of ducts with walls over-lying cell layer **G** *Vernonia echioides*, ﻿part of corolla lobe from dried herbarium specimen showing some short-stalked capitate glands **H** *Vernonia incana*, part of corolla lobe from dried herbarium specimen.

A unifying anatomical structure of the three related genera in the typical element of the tribe Vernonieae is found in the study of the corolla lobes of the group. The corolla lobes of *Vernonia* are filed with multiple parallel ducts containing resin or oils of some type (Fig. 1a–d). These ducts were first noted in the related genus *Trepadonia* H.Rob., and they were illustrated crudely with a photomicrograph in the paper describing that genus (Robinson 1994). Subsequently, during casual observations during systematric work, the ducts were seen in typical *Vernonia* and in numerous species of the closely related *Vernonanthura*. In the latter genus the ducts were often observed in dried specimens under the dissecting microscope. The ducts were represented schematically in the plates of generic charactertistics in the study of American Vernonieae by Robinson (1999b).

All initial observations of the ducts were in material mounted in Hoyer’s Solution, and no contents of the ducts were observed. It was when some material was observed microscopically in water mounts that resin or mucilage was seen in the ducts. Given the potential taxonomic importance of the ducts, a first determined effort is made here to provide a proper illustration of the structure.

## Methods

Material examined was from dried specimens in the US National Herbarium (US) plus fresh material of *Vernonia noveboracensis* (L.) Michx., the type species of the genus *Vernonia* Schreb., collected on Roosevelt Island and placed in FAA. Herbarium material studied included *Vernonia noveboracensis*, *Vernonanthura brasiliana* (L.) H.Rob., the type species of *Vernonanthura*, and the two species, *Vernonia echioides* Less., and *Vernonia incana* Less., the latter two species that until the present have been the only South American species retained in the genus *Vernonia*. For purposes of the study, four additional species of Vernonia were examined from dried material, *Vernonia acaulis* (Walt.) Gleason, *Vernonia baldwinii* Torr., and *Vernonia blodgettii* J.K.Small all from eastern North America, and *Vernonia karwinskiana* DC. From Oaxaca, Mexico. A final collection was of an unvouchered collection of *Vernonia angustifolia* Michx. from a garden in Arlington, Virginia, and placed directly on a microscope slide (See Appendix for citations).

For purposes of microtome sectioning, living material of *Vernonia noveboracensis* was field collected and fixed in formal-aceto-alcohol (FAA 1:1:18). Flower heads were subdivided, dehydrated with 2-2 dimethoxypropane (DMP) (Postek and Tucker 1976), then infiltrated and embedded in paraffin. Serial sections were made at 5 μm, stained with buffered Toluidine blue O (Sakai 1973) and mounted in Lipshaw’s synthetic mounting resin. Unopened buds were used to avoid the recurved condition of the lobes in opened florets. Placement of bud material in the mounting resin was uncertain since the florets had five lobes that were themselves somewhat curved. Cross-sections were easily obtained. Longitudinal sections were by chance.

For views of unsectioned material, corolla lobes were taken from fixed material and from dried herbarium specimens and one freshly collected specimen for whole mount observation.

Images (photomicrographs) were captured in bright field or Nomarski (DIC) using a Zeiss Standard 16WL microscope, a Zeiss Universal Research microscope or a Zeiss Axiophot equipped with a Retiga 1300i digital camera (Q Imaging Corp.) and an image acquisition and processing system capable of live tiling and live EDF (extended depth of field) by MediaCybernetics/ImageProPlus 7.0.

The material from dried herbarium specimens was remoistened in water and mounted on microscope slides in water to preserve any resin or mucilage that might be in the ducts.

## Results

There are two ways of showing the ducts in the corolla lobes of *Vernonia*. One is by sectioning the material, the other is by whole mounts of the corolla lobes placed under the microscope. Both methods have been used here, and both methods have unmistakably demonstrated the ducts.

Cross-sections of the corolla lobes of *Vernonia noveboracensis* clearly show the series of seven to twelve ducts in a lobe (Fig. 1A & B). The ducts are mostly surrounded by individual sheaths of cells, the same cells that form the partitions between the ducts that were obvious in the initial crude studies of the characteristic in *Trepadonia*. A longitudinal section (Fig. 1C) shows the ducts as elongate structures that lack cross-walls. The longitudinal sections of the corollas indicate that the ducts are not completely restricted to the corolla lobes, but that they begin below the lobes, especially along the main veins of the corolla. Both cross- and longitudinal sections show the ducts as empty; apparently Hoyer’s Solution and preservative both completely remove all resin or mucilage contents of the ducts.

The second method of viewing the ducts is the one that showed the structure in the paper describing *Trepadonia* (Robinson 1994). However, that preparation was in Hoyer’s Solution and retained no resin or mucilage in the ducts. The new views first used material that had been placed in preservative, and the ducts were also empty. Mounts of corollas in water, however, showed the ducts clearly with their chemical contents, and the presence of material in the ducts made the ducts more obvious.

Figures 1E & F show the lobes of *Vernonia angustifolia* and *Vernonanthura brasiliana* with resin or mucilage in the ducts. The duct contents show that multiple longitudinal ducts are present in both genera. The resin contents do not seem to appear in all the ducts of any lobe at the same time. The fresh material of *Vernonia angustifolia* obtained from garden plant shows lobe that has neither been dried nor treated with chemicals. Even here, the ducts seem to be only partially filled with resin. Observations of *Vernonia acaulis*, *Vernonia baldwinii*, *Vernonia blodgettii*, and *Vernonia karwinskii* were in water on slides simply to confirm the presence of the ducts in a broader representation of the genus. While the ducts can be seen once the observer is familiar with their appearance, they are not quite obvious enough to be used as anything more than a supplementary key character in taxonomic treatments. The character could be useful in separating *Vernonanthura* from *Critoniopsis* Sch.Bip. in the Andes, and *Vernonanthura* from the adventive *Gymnanthemum amygdalinum* (Del.) Sch.Bip. ex Walp. in Brazil.

The survey of the whole tribe for presence of multiple ducts has not been rigorous, but certainly on a par with the observations that led to the initial discovery of the ducts in *Trepadonia*, *Vernonanthura* and *Vernonia* (Robinson 1994). It remains to be seen what different kind of internal structure occurs in the corollas in other members of the Vernonieae. It can only be said that multiple longitudinal ducts have not been seen in any of the other genera. As an indication other possible specialization, at least one African species, still known as *Vernonia potamophila* Klatt, shows chambers of some kind in its lobes.

The nature of the chemical contents of the ducts remains unknown. The types of chemicals known from the Vernonieae include epoxy resins, but these have been found most notably in genera like *Stokesia* L’Hér. (Gunn and White 1974) and *Centrapalus* Cass. (Purdue et al. 1986, 1989), neither of which has the multiple longitudinal ducts in the corollas. Terpenoids are common in many Vernonieae (Herz 1977; Bohlmann and Jakupovic 1990), but their end products are not tolerated by Insects and Mammals or the primary tissues of the plants in which they are produced. For that reason, in the Asteraceae, the terpenoids are accumulated in special cells usually at the tips of glandular hairs such as those seen on the lobe of *Vernonia echioides* (Fig. 1G). Acetylenes are a stronger possibility, but they are not varied in Vernonieae where only pentaynenes have been reported (Bohlmann et al. 1973). In other tribes such as the Heliantheae, Acetylenes are highly varied and occur in ducts, and where they seem to have been credited with a mostly antifungal function that ceases with the death of the plant. These are the types of secondary metabolites most widely known to occur in the tribe Vernonieae, and the identity of the duct contents remains unresolved.

It seemed particularly useful to examine the corolla lobes of the two South American species that had thus far been retained in *Vernonia* on the basis of the pollen and habit of the plants. Their position in *Vernonia* was always in question because of their remote geographical locations in Argentina, Brazil, Paraguay and Uruguay. The material was chosen from herbarium specimens that showed no indication of being preserved in alcohol or formalin. As shown in the figures (1G, H) no trace of multiple longitudinal ducts was seen. The character of the ducts is here regarded as a defining feature of the generic group of *Vernonia*, *Vernonanthura* and *Trepadonia* and the lack of the feature in the two South American species, combined with the remote geographical location is considered strong evidence that they do not belong in *Vernonia*. DNA of the two species has not yet been sequenced, and their proper disposition remains to be determined.

## Appendix

### Specimens used in microscopic illustrations

*Vernonanthura brasiliana* (L.) H.Rob., Venezuela: Lara: vicinity of Barquisimeto, 4 Jan 1923, *José Saer 102* (US).

*Vernonia angustifolia* Michx., Arlington VA, corner of N. Calvert and 18^th^ Sts, unvouchered garden plant: Aug. 2015, *H. Robinson s.n*.

*Vernonia echioides* Less., Brazil: Paraná, São João, in paludosis, 21 Mar. 1910, *P. Dusén 9370* (US).

*Vernonia incana* Less., Paraguay: Dept. Neembucu, Dist. Yataity, Esterito, Jan 1975, *M.A.Walter 118* (US).

*Vernonia noveboracensis* (L.) Michx., Washington, D.C., Roosevelt Island: 19 Aug. 2014, *H. Robinson 14-1* (US).
